# Sanitary safety of the 2021 French Intensive Care Society medical conference: a case/control study

**DOI:** 10.1186/s13613-022-00986-x

**Published:** 2022-02-11

**Authors:** SRLF Trial Group, Pierre-Yves Boelle, Guillaume Decormeille, Bertrand Hermann, Nicholas Heming, Gwenaelle Jacq, Toufik Kamel, Jean-Baptiste Lascarrou, Eric Maury, Laurent Papazian, Gael Piton, Laurent Poiroux, Julien Ramillon, Anahita Rouze

**Affiliations:** Service de Médecinadditie Intensive Réanimation, Centre Hospitalier Universitaire, 44093 Nantes Cedex 1, France

**Keywords:** Mass gathering event, SARS-CoV-2, COVID-19, Transmission, Infectious disease, Prevention

## Abstract

**Background:**

In-person mass gathering events (MGE) are returning after a period of restrictions, yet few prospective scientific evaluations of their safety are available.

**Methods:**

Prospective observational study, including both attendees of the French Intensive Care Society (FICS) annual meeting held in Paris between June the 9th and June the 11th, 2021 and matched controls (healthcare professionals who stayed in the ICU during the conference). SARS-CoV-2 lateral flow test was performed on day 7. Follow-up occurred until day 21.

**Results:**

Out of the 1824 healthcare professionals attending the congress (all of which fulfilled legal requirements: 7 days or more following a second dose of vaccine or a negative PCR test performed within less than 72 h), 520 (28.5%) agreed to participate. Follow-up data were received for 216 (41.5%) out of the 520 included attendees, and for 191 matched controls. No positive SARS-CoV-2 lateral flow test was reported in the attendees or in the matched controls. The probability of SARS-CoV-2 infection during the MGE was less than 1.7% in the attendees (95% confidence interval [0;1.7%]), less than 2% in the controls (95%CI [0;2%]) and the difference in probabilities of infection was less than 1.9% (95% CI [0;1.9%]).

**Conclusion:**

During a low incidence period, in this population of congress attendees screened for SARS-CoV-2 by a lateral flow test at day 7, no positive cases could be documented, no concomitant infection occurred in the matched controls; suggesting no extra risk of infection during the MGE.

*Trial Registration*: ClinicalTrial.gov, #NCT04918160.

**Supplementary Information:**

The online version contains supplementary material available at 10.1186/s13613-022-00986-x.

## Introduction

Non-pharmacological interventions aimed at preventing SARS-CoV-2 transmission include contact tracing and quarantine of cases, up to lockdowns when rates of infection are higher as well as physical distancing; which includes limiting the occurrence of mass gathering events (MGE), [1]. Indeed, several outbreaks of SARS-CoV-2 were associated with MGE [2], linked to the attendance of asymptomatic carriers who may nevertheless transmit the virus. Indeed, SARS-CoV-2 transmissibility begins 2 to 3 days before symptom onset; nearly half of all transmissions arise through contact with asymptomatic individuals [3]. Additionally, reverse transcriptase-polymerase chain reaction (RT-PCR) test results are delayed, making it an impractical tool for mass testing strategies prior to a MGE. Alternative point-of-care diagnostic methods include antigen-detecting rapid diagnostic tests. Lateral flow tests (LFT) detect the nucleocapsid protein antigen of SARS-CoV-2 and provide results within 30 min. LTF may be self-administered at home, enabling the rapid identification of people infected by the virus.

Despite improved capacities of detecting SARS-CoV-2 infections, few prospective assessments of the sanitary safety of mass gathering events (MGE) have been performed. In a pivotal study conducted in Spain, out of 465 spectators of a live music event, 13 (3%) subjects were found to be positive for SARS-CoV-2 antigen testing [4]. The same team found in a subsequent study, found that out of 5000 subjects willing to spectate a live music event, 6 were tested positive for SARS-CoV-2 [5]. However, these observations occurred prior to mass vaccination of the population. In France, Delaugerre et al. in a randomized controlled trial with almost 4000 attendees, found than participation in a large, indoor, live gathering without physical distancing was not associated with increased SARS-CoV-2 transmission risk, provided a comprehensive preventive intervention was implemented [6].

Among MGE, medical conferences are of special interest because they bring together healthcare workers who are on the frontline of the fight against COVID-19. Said healthcare workers are exposed on a day-to-day basis to SARS-CoV-2 both in their COVID-19 units and out of hospital. Additionally, healthcare workers are essential to provide adequate care for COVID-19 infected patients; sickness leave due to SARS-CoV-2 exposure or infection may weaken already overstretched healthcare system. This is especially true in intensive care units (ICU) [7]. The sanitary safety of medical congresses is thus of utmost importance.

To assess potential transmission of SARS-CoV-2 during the French Intensive Care Society annual meeting, we conducted a prospective study with systematic LFT tests performed by attendees and controls 7 days after the event. This event took place in a context where vaccination and/or negative SARS-CoV-2 testing were required for attendance.

## Patients and methods

### Study design

We conducted a prospective observational study, including both attendees to the French Intensive Care Society (FICS/SRLF) annual meeting held in Paris between June 9 and June 11, 2021 and matched controls. Indeed, given the specific exposure of ICU healthcare professionals, inclusion of a control grouped seemed mandatory. Matched controls were colleagues of attendees who stayed in ICU throughout the medical conference. Data were collected during the meeting and up to day 21 after the meeting. This report follows the STROBE guidelines [7].

### Requirements to attend the congress

French regulation in place at the time of congress mandated each attendee to comply with at least one the following requirements:

- 7 days after 2nd administration for dual dose vaccines (Pfizer©, Moderna©, AstraZeneca©);
- 28 days after administration for single dose vaccines (Janssen/Johnson & Johnson©);
- 7 days after administration of the vaccine for people who had previously contracted COVID-19 (only 1 injection) more than 6 months ago;
- Recovery from COVID-19 attested by the result of a positive SARS-CoV-2 RT-PCR or antigenic test dating from at least 15 days and less than 6 months (associated with a limited risk of reinfection with COVID-19) [8].
- Proof of a negative SARS-CoV-2 RT-PCR or antigenic detection test performed within less than 48 h.

Other mitigation strategies during the congress were implemented and included: availability of hand sanitizers, mandatory wearing of surgical face masks and adequate ventilation of all congress areas. Ventilation of congress areas was assessed by measuring average carbon dioxide (CO2) levels using a CO2 Monitoring device Air Therm (La Mode, London, UK).

### Participant selection

Inclusion criteria for the attendees were:

- Healthcare professional,
- Absence of COVID-19 symptoms over the 2 weeks prior to inclusion,
- Not being contact of a case of COVID-19 over the 2 weeks prior to inclusion,
- Attending at least one day of the FICS annual meeting (June 9–11 2021).

Exclusion criteria were:

- Non-healthcare professionals,
- Refusal to participate,
- Guardianship or tutorship,
- Absent affiliation to the French social security.

Controls were recruited by the attendees. The same inclusion and exclusion criteria were applied to controls except that they did not participate to the annual meeting. Controls were matched to attendees in terms of gender, age (< or > 40 years), profession (medical doctor, nurse, nurse assistant, other) and vaccine status (complete, partial, non-vaccinated).

### Self-administered LFT SARS-CoV-2 tests

LFT for SARS-CoV-2 aims to detect infection by recognizing viral proteins. Most LFT use specific labeled antibodies attached to a nitrocellulose matrix strip to capture viral antigens. The Flowflex SARS-CoV-2 Antigen Rapid Test is a lateral flow chromatographic immunoassay for the qualitative detection of the nucleocapsid protein antigen from SARS-CoV-2 in nasal and nasopharyngeal swab specimens directly from individuals who are suspect of COVID-19. Successful binding of the antibodies to the antigen is visually detected through the appearance of a line on the matrix strip. The Flowflex SARS-CoV-2 Antigen Rapid test provides results within 30 min.

LFTs were freely provided at the time of inclusion. 7 ± 1 days after the attendee’s last day of attendance, the attendee and his control were asked to perform a LFT.

### Data collection

Standardized forms were used to record the following data: participant’s baseline characteristics (age, gender, height, weight) and COVID-19 status (previous COVID-19 infection and date of occurrence), risk factors of developing a severe form of COVID-19 (see Additional file 4: Table S1), anti-SARS-CoV-2 vaccination status (number of doses, date of injection, type of vaccine), results of the day 7 self-administered LFT and occurrence of COVID-19 symptoms and/or need of medical assistance for a COVID-19 infection over a three week period following the congress. Follow-up was obtained at day 21 for each participant and control.

### Ethics

The study was approved by French Infectious Disease Society ethics committee (*CERMIT N° COVID 2021-08*) and was registered on ClinicalTrials.gov (#NCT03600181). Written information was delivered to all participants. All participants entered the study following oral consent.

### Outcomes

The primary endpoint was the prevalence of attendees with a positive COVID-19 self-administration LFT at day 7 of participation to the congress.

Secondary objectives were:

- COVID-19 prevalence among controls at Day 7,
- Proportion of congress attendees with COVID-19 symptoms by Day 21,
- Proportion of congress attendees requiring COVID-19-related appointment with a general practitioner by Day 21,
- Proportion of congress attendees with COVID-19-related emergency department visits by Day 21,
- Proportion of conference attendees with COVID-19-related hospitalization by Day 21.

### Sample size

Given the exploratory nature of our study, we did not calculate a sample size. We aimed at including a convenience sample of 500 attendees and 500 controls (1000 participants).

### Statistical analysis

Qualitative variables were described as number (%) and quantitative variables as mean ± SD if normally distributed and as median [25th–75th percentile] otherwise. Matching factors between attendees and controls were compared by means of Fisher tests. All tests were two-tailed with a significance level of 0.05.

Confidence interval for the probabilities of being infected for attendees and controls was performed by means of one-tailed exact 95% confidence interval for binomial proportions. For the difference in proportions, we used the simple “add 2 success, add 2 failures” method [9].

All statistical analyses were performed using R statistical software version 4.0.3.

## Results

### Baseline characteristics

Out of the 1824 healthcare workers attending the congress, 520 (28.5%) agreed to participate. Baseline characteristics and comparison with attendees who did not participate are depicted in Table 1. Regional origins of both groups are presented on Additional file 1: Figure S1. Briefly, participants were mainly female and nurses and we observed some regional disparities.

**Table 1:** Comparison of included and not included participants of the congress

	Included (n = 520)	Not included (n = 1304)	P
Gender, male, n (%)	229 (44)	649 (49.8)	0.031
Professional category, n (%)			< 0.001
Doctor or resident	281 (54)	700(53.7)	
Nurse	153 (29.4)	319(24.5)	
Assistance nurse	17 (3.3)	36(2.8)	
Physiotherapists	34 (6.5)	60(4.6)	
Others	35 (6.7)	189(14.5)	
Age, years, mean ± SD	38.2 ± 11.0	39 ± 11.9	0.26
Country, n (%)			0.654
France	406 (78.1)	1208 (92.6)	
Others*	26 (5.0)	88 (6.7)	
Not known	88 (16.9)	8 (0.6)	
French administrative region, n (%)			< 0.001
Auvergne-Rhône-Alpes	45 (8.7)	109 (8.3)	
Bourgogne-Franche-Comté	16 (3.1)	43 (3.2)	
Bretagne	2 (0.4)	20 (1.5)	
Centre-Val de Loire	10 (1.9)	34 (2.6)	
Grand Est	40 (7.6)	81 (6.2)	
Hauts-de-France	43 (8.2)	81 (6.2)	
Ile-de-France	139 (26.7)	613 (47.0)	
Normandie	20 (3.8)	58 (4.4)	
Nouvelle-Aquitaine	28 (5.3)	75 (5.7)	
Occitanie	17 (3.2)	53 (4.0)	
Outre-mer	4 (0.7)	10 (0.7)	
Pays de la Loire	30 (5.7)	56 (4.3)	
Provence-Alpes-Côte d’Azur	36 (6.9)	47 (3.6)	
Unknown	90 (17.3)	24 (1.8)	

*Some participants came from French-speaking countries such as Belgium or Switzerland (not detailed in case report form)

The percentages may not total 100 because of rounding. Missing data not included in statistical analysis

### Follow-up

**Fig. 1:**
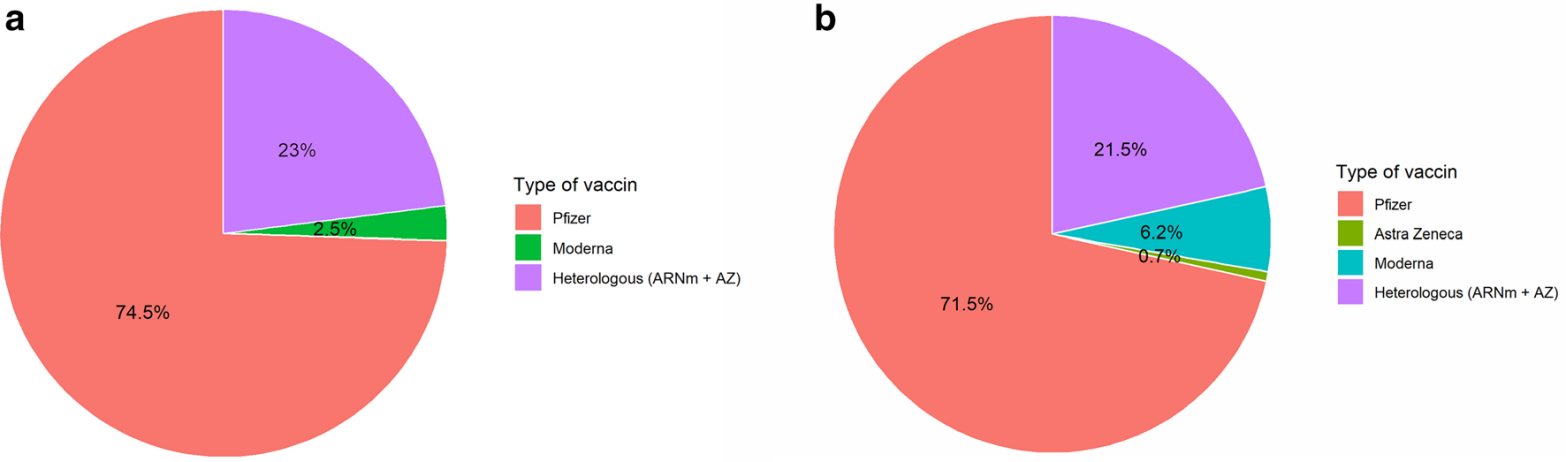
Distribution of vaccine type.** a** Attendees (n=200, 16 patients with missing value).** b** Controls (n=191, 15 patients with missing value)

Follow-up data were obtained for 216 (42%) out of 520 participants, and for 191 controls. Characteristics of attendees and controls were well matched except for the profession, with a higher percentage of medical doctors among the attendees as compared to nurses (i.e., less medical doctors succeed in recruiting controls) (Table 2, Fig. 1 and Additional file 2: Figure S2).

**Table 2:** Comparison of attendees and controls

	Attendees (N = 216)	Controls (N = 191)	P
Gender, n, %			0.84
Male	101 (46.8)	88 (46.1)	
Female	109 (50.5)	99 (51.8)	
Missing	6 (2.8)	4 (2.1)	
Profession			0.001
Doctor	131 (60.6)	100 (52.4)	
Nurse	55 (25.5)	46 (24.1)	
Assistant nurse	2 (0.9)	9 (4.7)	
Physiotherapist	19 (8.8)	16 (8.4)	
Others	2 (0.9)	15 (7.9)	
Missing	7 (3.2)	5 (2.6)	
Age			0.47
< 50 years	160 (74.1)	149 (78.0)	
≥ 50	49 (22.7)	38 (19.9)	
Missing	7 (3.2)	4 (2.1)	
History of COVID-19			*
No	216 (100)	171 (89.5)	
Yes	0 (0)	2 (1.0)	
Missing	0 (0)	18 (9.4)	
Vaccinal status (number of dose) ^$^			0.77
0 dose	8 (3.7)	10 (5.2)	
1 dose	38 (17.6)	32 (16.8)	
2 doses	162 (75.0)	145 (75.9)	
Missing	8 (3.7)	4 (2.1)	
Vaccinal status (binary)			0.90
Complete	161 (74.5)	144 (75.4)	
Incomplete	38 (17.6)	32 (16.8)	
Missing	17 (7.9)	15 (7.9)	
BMI			0.22
BMI < 18.5	2 (0.9)	4 (2.1)	
18.5 ≤ BMI ≤ 25	149 (69.0)	116 (60.7)	
25 ≤ BMI ≤ 30	44 (20.4)	49 (25.7)	
BMI > 30	14 (6.5)	18 (9.4)	
Missing	7 (3.2)	4 (2.1)	
Vulnerability			0.52
No	199 (92.1)	157 (82.2)	
Yes	11 (5.1)	12 (6.3)	
Missing	6 (2.8)	22 (11.5)	

*BMI* body mass index

^*^Not enough events for statistical comparison

^$^Legal requirement to attend congress was 7 days after 2 doses or PCR test less than 2 days, explaining medium rate of 2 doses vaccination status

Missing data not included in statistical analysis

### Primary outcome

No positive COVID-19 LFT was reported in the attendee’s group. The 95% confidence interval estimate of the probability of infection during the congress for attendees ranged between 0 and 1.7%.

### Secondary outcomes

No positive COVID-19 LFT was reported in the control group either, leading to an estimated 95% confidence interval probability of contamination during the congress between 0 and 2%. Accordingly, we computed the confidence interval for the difference in probability of infection between attendees and controls using the “add 2 success and add 2 failures”, yielding a difference in the range [− 1.9;1.9%] (95% confidence interval). Secondary outcomes are presented in Table 3. No significant difference between attendees and controls were found except for the probability that the LFT was performed (98.1% *vs* 80.6%; P < 0.001). CO2 levels throughout the congress were median [IQR] 594 [561–687] ppm during the event (Additional file 3: Figure S3).

**Table 3:** Secondary outcomes

	Attendees (N = 216)	Controls (N = 191)	P value
Symptoms at day 7, n (%)			> 0.99
No	214 (99.0)	172 (90.0)	
Yes	1 (0.5)	0 (0.0)	
Missing	1 (0.5)	19 (10.0)	
AutoLTF performed, n (%)			< 0.001
No	4 (1.9)	18 (9.4)	
Yes	212 (98.1)	154 (80.6)	
Missing	0 (0.0)	19 (9.9)	
Symptoms until day 21, n (%)			0.50
No	214 (99.1)	175 (91.6)	
Yes	2 (0.9)	0 (0.0)	
Missing	0 (0.0)	16 (8.4)	
Additional test performed at day 21, n (%)			0.70
No	198 (91.7)	163 (85.3)	
Yes	18 (8.3)	12 (6.3)	
Missing	0 (0.0)	16 (8.4)	
Need for medical consults until day 21, n (%)			–
No	216 (100)	173 (90.6)	
Yes	0 (0.0)	0 (0.0)	
Missing	0 (0.0)	18 (9.4)	

Missing data not included in statistical analysis

## Discussion

We found that, in the current area of highly effective vaccines, SARS-CoV-2 transmission during a medical congress was extremely low and similar in controls who did not attend the conference.

A recent review of published studies focusing on the impact of mitigation strategies during the COVID-19 pandemic [10] concluded that “there is currently limited evidence on the effectiveness of measures to prevent SARS-CoV-2 transmission at mass gatherings”. Our results are in line with previous studies on the safety of mass gathering events while respecting physical distancing and simple measures of hygiene. In an earlier study, Flury et al. included 196 participants out of the 365 attendees of the Swiss Societies of Infectious Diseases and Hospital Hygiene congress [11]. The authors found that 5 participants presented a positive SARS-CoV-2 serology at follow-up, all whom were already positive at baseline. In the study by Revollo et al. [4], out of the 495 participants attending a music event, none presented a positive PCR at day 8, resulting in a Bayesian estimate for the incidence between the exposed and the non-exposed of − 0·15% (95% CI – 0·72 to 0·44). In our study, the frequentist estimate for the difference in incidence between the participants and the controls ranged between − 2 to 2%.

In addition to these results, our study highlights several challenges encountered in epidemiological studies assessing SARS-CoV-2 transmission. First, participation in a scientific evaluation of transmission is challenging, even in a population of healthcare workers confronted to COVID-19 since the beginning of the pandemic. Indeed, only around 30% of healthcare workers attending the conference agreed to be included and less than half of these self-administered their LFT at day 7 and reported their status after the congress. This low feed-back from participants in the context of self-delivered COVID-19 testing must be anticipated especially for self-funded research study. Second, we found that the recruitment of controls by participants allowing a fair comparison of infectious disease contamination rates is possible for participants who are healthcare workers in departments highly involved in clinical research [12], with almost as many controls as attendees recruited. We also showed that COVID-19 LFT can be self-performed by participants without major technical challenge. These tests could thus be used to mitigate the risk of COVID-19 transmission in other settings. Indeed, although LFT are less sensitive than COVID-19 PCR and despite commercial kits displaying variable performance, their cost is reduced compared to PCR, greatly facilitating mass testing. The sensitivity of the LFT used in this study was 94.5%, i.e., one of the best tests available at the time of the study [13]. In a study of 5,869 asymptomatic adults, Garcia-Finana et al. found that LFT were likely to detect a minimum of 3/5 and at most 998 out of every 1,000 subjects with a positive RT-qPCR test result and a high viral load [14]. We chose to perform LFT at day 7, since it was the best time window to detect SARS-CoV-2 transmission [15]. Our follow-up rate was 42% (216/520), similar to previous reports, i.e., between 13 and 66% [16]. Lastly, CO_2_ levels measured during the event were broadly lower than recommended (between 600 to 1500 parts per million) [17, 18] indicating that the venue was sufficiently ventilated.

In addition, limitations due to the low rate of attendee recruitment and feed-back from participants, and the generalizability of our study must be discussed. First, our study was underpowered to detect a small but potentially clinically relevant difference due to the relatively low incidence of COVID-19 cases in France (40 cases/100.000) and Great Paris area (65 cases/100.000) at the time of the congress. Second, at the time of the congress, the SARS-CoV-2 alpha variant was still dominant; our results may therefore not be true faced with delta or any other highly transmissible variant. Third, our study must be interpreted in the context of this specific mass event: young age of the participants and high rate of participants within 6 months of a complete vaccination scheme. Last, absence of randomization or of stratification by region or by profession, limit causal inference between congress attendance and risk of SARS-CoV-2 transmission.

## Conclusion

In our study of a medical congress, screening for SARS-CoV-2 transmission by LFT at day 7, identified no positive cases among congress attendees or matched controls. In the context of low overall SARS-CoV-2 incidence and a predominant alpha variant, we found than mass event gathering can be safely performed if adequate mitigations procedures are applied before and during the congress.

## Supplementary Information

**Additional file 1: Figure S1**. Graphic representation of attendees and participants.

**Additional file 2: Figure S2**. Distribution of vaccine type for fully vaccinated.

**Additional file 3: Figure S3**. Evolution of CO2 in the meeting area.

**Additional file 4: Table S1**. Vulnerability criteria.

## Data Availability

The study data will be made available upon reasonable request to the Research Commission of the French Intensive Care Society.
