# Supplementary figures and images for: Sanitary safety of the 2021 French Intensive Care Society medical conference: a case/control study
Source: Ann Intensive Care. 2022 Feb 11;12:11. doi: 10.1186/s13613-022-00986-x (PMC8831193; doi:10.1186/s13613-022-00986-x)

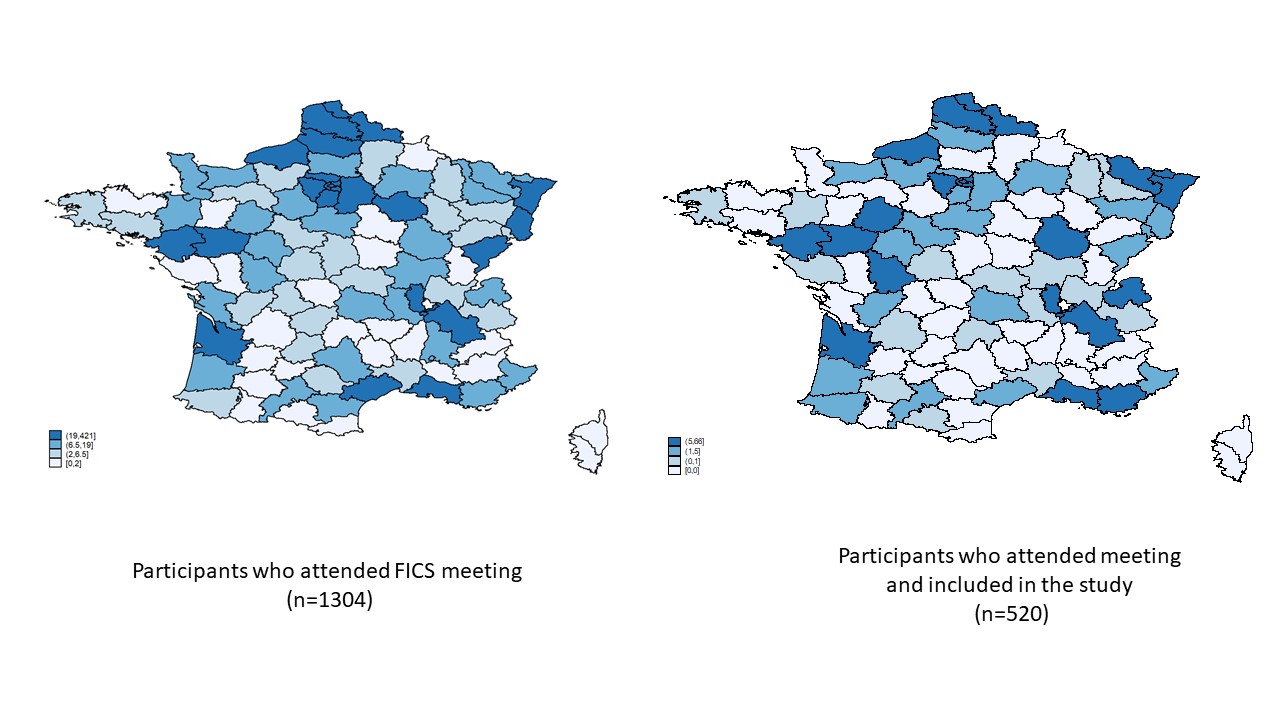

Supplement: Supplementary file 1 — Additional file 1: Figure S1. Graphic representation of attendees and participants. [file 13613_2022_986_MOESM1_ESM.docx]
