# Supplementary material for: Sanitary safety of the 2021 French Intensive Care Society medical conference: a case/control study
Source: Ann Intensive Care. 2022 Feb 11;12:11. doi: 10.1186/s13613-022-00986-x (PMC8831193; doi:10.1186/s13613-022-00986-x)
Supplement: Supplementary file 2 — Additional file 2: Figure S2. Distribution of vaccine type for fully vaccinated. [file 13613_2022_986_MOESM2_ESM.docx]

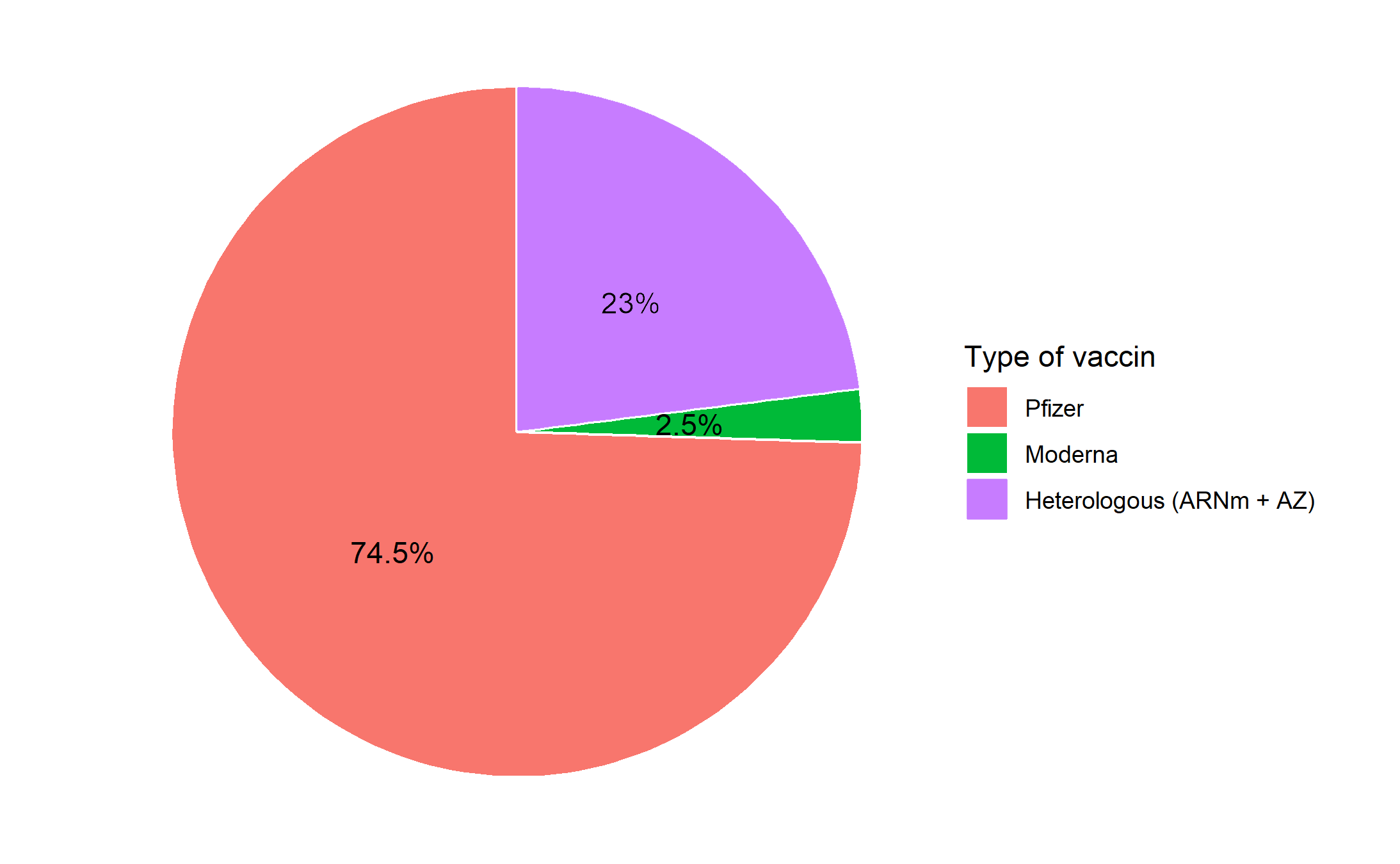

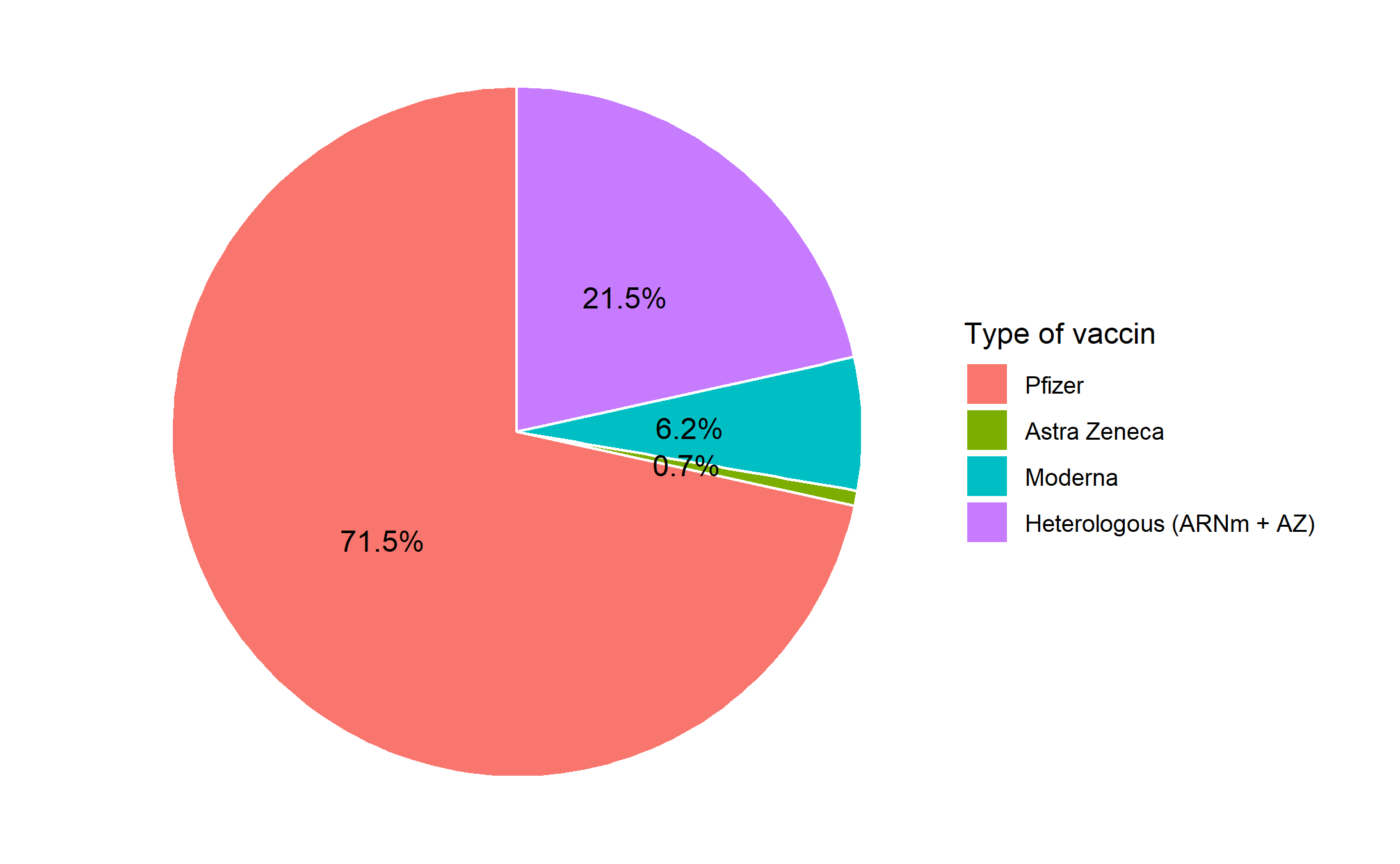


Distribution of vaccine type for controls with complete vaccination scheme

(n=144)

Distribution of vaccine type for attendees with complete vaccination scheme

(n=161)
