# Supplementary material for: Sanitary safety of the 2021 French Intensive Care Society medical conference: a case/control study
Source: Ann Intensive Care. 2022 Feb 11;12:11. doi: 10.1186/s13613-022-00986-x (PMC8831193; doi:10.1186/s13613-022-00986-x)
Supplement: Supplementary file 3 — Additional file 3: Figure S3. Evolution of CO2 in the meeting area. [file 13613_2022_986_MOESM3_ESM.docx]

CO2 detection was performed with Air Therm device (Therm La Mode, London, United Kingdom).
