# Supplementary material for: Sanitary safety of the 2021 French Intensive Care Society medical conference: a case/control study
Source: Ann Intensive Care. 2022 Feb 11;12:11. doi: 10.1186/s13613-022-00986-x (PMC8831193; doi:10.1186/s13613-022-00986-x)
Supplement: Supplementary file 4 — Additional file 4: Table S1. Vulnerability criteria. [file 13613_2022_986_MOESM4_ESM.docx]

Risk factors of developing a severe form of COVID-19 defined following French guidelines on January 11, 2021:

- Be 65 years of age or older;
- Have a history of cardiovascular disease: complicated hypertension (with cardiac, renal and vascular-cerebral complications), history of stroke or coronary artery disease, heart surgery, NYHA stage III or IV heart failure;
- Have an unbalanced diabetes or one with complications;
- Present a chronic respiratory pathology likely to decompensate during a viral infection: obstructive bronchial pneumonia, severe asthma, pulmonary fibrosis, sleep apnea syndrome, cystic fibrosis in particular; Present a chronic renal insufficiency dialyzed;
- Be suffering from cancer undergoing treatment (excluding hormone therapy); Being obese (body mass index (BMI) > 30 kgm2); Have cirrhosis at least stage B of the Child Pugh score;
- Have a major sickle cell syndrome or a history of splenectomy; Be in the third trimester of pregnancy;
- Have congenital or acquired immunodepression: drug-induced: cancer chemotherapy, immunosuppressive treatment, biotherapy and/or corticosteroid therapy at immunosuppressive dose; uncontrolled HIV infection or with CD4 < 200/mm3; following a solid organ or hematopoietic stem cell transplant; related to a hematologic malignancy under treatment;
- Having motor neuron disease, myasthenia gravis, multiple sclerosis, Parkinson's disease, cerebral palsy, quadriplegia or hemiplegia, primary malignant brain tumor, progressive cerebellar disease, or a rare disease.
